# Transdentinal effects of flavonoid-based primers applied to caries-affected dentin on pulp cells

**DOI:** 10.1007/s00784-025-06693-8

**Published:** 2025-12-08

**Authors:** Beatriz Ometto Sahadi, Igor Paulino Mendes Soares, Chloe Gifford, Caroline Anselmi, Josimeri Hebling, Marcelo Giannini, Marco C. Bottino

**Affiliations:** 1 Department of Restorative Dentistry, Piracicaba Dental School, University of Campinas, Piracicaba, SP Brazil; 2 Department of Cariology, Restorative Sciences, and Endodontics, University of Michigan School of Dentistry, 1011 N. University (Room 2303), Ann Arbor, MI 48109 USA; 3 Department of Dental Materials and Prosthodontics, School of Dentistry, São Paulo State University UNESP, Araraquara, SP Brazil; 4 Department of Morphology and Pediatric Dentistry, School of Dentistry, São Paulo State University UNESP, Araraquara, SP Brazil; 5 Department of Biomedical Engineering, College of Engineering, University of Michigan, Ann Arbor, MI USA

**Keywords:** Flavonoids, Toxicity, Odontoblasts, Dental caries, Dentin

## Abstract

**Objective:**

To examine the transdentinal response of odontoblast-like cells (MDPC-23), dental pulp stem cells (DPSC), and macrophages to flavonoid-based primers applied to caries-affected dentin.

**Materials and methods:**

Primers were prepared from 20 mM of Naringin, Kaempferol, or Baicalein in 20% ethanol. Caries-affected dentin discs (*N* = 56) were produced using a microcosm biofilm model and mounted in artificial pulp chambers with MDPC-23 cells seeded on the pulp side. Occlusal surfaces (*n* = 8) were etched with 35% phosphoric acid, rinsed, and blot-dried, followed by application of ultrapure water (negative control, NC), 20% ethanol (solvent control, SC), flavonoid primers, or 29% hydrogen peroxide (positive control, PC). After 24 h, cell viability and morphology (scanning electron microscopy, SEM) were evaluated. Extracts obtained by transdentinal diffusion were applied to DPSC and MDPC-23 cells for up to 7 days to assess cell viability and mineralization, and to macrophages (RAW 264.7) for reactive oxygen species (ROS) production. Data were analyzed using ANOVA and Sidak’s test (α = 5%).

**Results:**

None of the flavonoid primers reduced MDPC-23 viability, while PC and phosphoric acid showed significant differences from NC. SEM images revealed altered cell morphology in acid-etched groups. After 7 days, primers slightly increased DPSC viability, maintained MDPC-23 viability, promoted mineralization, and reduced ROS.

**Conclusions:**

Flavonoid-based primers applied to caries-affected dentin were non-cytotoxic, stimulated mineralization in pulp cells, and lowered oxidative stress in macrophages.

**Clinical relevance:**

Flavonoid primers could support pulp health during dentin pretreatment, providing a conservative strategy for managing caries-affected dentin and reducing the risk of pulp irritation.

## Introduction

Adhesion to the dentin substrate remains a challenge in restorative dentistry, particularly when it is compromised by caries or when phosphoric acid is applied before the adhesive [1, 2]. This complexity is primarily due to the intrinsic characteristics of dentin, such as its collagen-rich organic composition and the presence of the dentinal fluid, which can hinder the infiltration and polymerization of adhesives [3, 4]. In addition to structural modifications of collagen caused by cariogenic bacteria, both scenarios lead to the degradation of collagen fibrils due to the activation of matrix metalloproteinases (MMPs). These endogenous enzymes, which are more abundant in caries-affected dentin [5], compromise the long-term stability of the hybrid layer [2, 4, 6]. Furthermore, adhesive resins may undergo hydrolytic degradation, promoted by water absorption and the leaching of unpolymerized monomers, ultimately reducing bond strength [7]. The interaction of these factors contributes to restorative failures, such as marginal leakage and secondary caries, which diminish the longevity of composite resin restorations [3, 4].

Several strategies have been studied to mitigate the damage caused by enzymatic degradation and to increase or stabilize the dentin bonding [8–10]. One such approach involves the use of experimental *primer* solutions based on natural products [10, 11]. The application of flavonoid-based experimental *primers* has been investigated as a clinical strategy in adhesive bonding procedures, aiming to enhance dentin biomodification, improve the stability of dentin-resin bonding, and consequently increase the longevity of restorations over time [10, 11]. In this context, flavonoid-based primers are proposed as a dentin biomodification step when the remaining dentin thickness is sufficient for their safe use, in line with previous studies using natural collagen cross-linkers [11, 12]. Since placing adhesive systems in deep cavities without cavity liners or bases remains controversial [13], the main goal of using flavonoid-based primers is to enhance the quality of the caries-affected substrate by stabilizing the dentin matrix, regardless of the material applied.

Flavonoids, due to their antioxidant properties [14], have been shown to effectively inhibit the activity of matrix metalloproteinases (MMPs), which are responsible for the degradation of collagen fibrils within the hybrid layer [15]. Furthermore, they can enhance the mechanical properties of dentin by promoting cross-linking of collagen fibers, which contributes to increased resistance to hydrolytic degradation [10]. Previous studies have shown that flavonoids demonstrated excellent performance in reducing the degradation of the hybrid layer, contributing to the preservation of bonding interfaces [8, 10, 11]. Recent studies have highlighted their potential in improving the longevity of composite resin restorations, particularly in caries-affected dentin, by stabilizing the dentin-adhesive interface [12] and preventing the development of secondary caries. Meanwhile, flavonoids may play a significant role in enhancing bond strength in caries-affected dentin, a challenging substrate due to its altered composition [10–12]. In dentin demineralized by cariogenic bacteria, the hydroxyapatite crystals are incompletely dissolved, the organic matrix is completely or partially degraded, and the dentinal tubules may exhibit an increased diameter, as there is a reduced amount of mineralized peritubular dentin [16, 17]. The modification of the collagen structure of the dentin caused by demineralization increases the permeability of the tissue [18], facilitating the diffusion of the primer. This increased primer diffusion can be exacerbated with phosphoric acid etching, which allows deeper penetration of primers and adhesive materials into dentin, potentially reaching pulp cells. This may have significant implications for dentin-resin bond strength and the prevention of pulp damage in composite resin restorations.

In addition to their effects on dentin biomodification, flavonoids have demonstrated antioxidant and anti-inflammatory properties [14], which may modulate pulp immune responses [19]. Macrophages play a key role in the innate immune-inflammatory events associated with the regenerative responses of pulp cells during tertiary dentinogenesis and are major contributors to reactive oxygen species (ROS) release and subsequent oxidative stress at the dentin-pulp interface [20]. Therefore, evaluating the potential of flavonoid-based primers to modulate ROS production provides valuable insight into possible immunomodulatory benefits associated with their use in deep cavities. Moreover, several flavonoids have been shown to influence the odontogenic potential of dental pulp stem cells (DPSCs), promoting mineralized nodule formation and upregulating markers involved in dentin–pulp complex repair [19, 21]. Taken together, these biological mechanisms suggest that flavonoid-based primers may exert effects not only on dentin substrate quality but also on pulp cell behavior and inflammatory modulation, which justifies the investigation of cytocompatibility, mineralization potential, and ROS production in the present study.

Despite the significant advantages associated with restorative procedures, the potential cytotoxic or bioactive effects of these flavonoid-based primers on dental pulp stem cells (DPSCs) and odontoblast-like cells (MDPC-23) remain unknown, especially when applied to caries-affected dentin. Understanding their biological impact is crucial, as direct or indirect exposure to these compounds could influence cell viability, proliferation, and differentiation ability, potentially affecting pulp tissue health and the long-term success of adhesive restorations. Thus, this study aimed to evaluate the biological response of dental pulp stem cells (DPSCs) and odontoblast-like cells (MDPC-23) to the application of flavonoid-based primers on caries-affected dentin, as well as to assess the anti-inflammatory potential of these solutions in macrophages through reactive oxygen species (ROS) analysis. The null hypotheses of this study were that the flavonoid-based experimental primers (1) would not affect the viability of pulp cells, (2) would not promote the formation of mineralized nodules, and (3) would not reduce the production of ROS in macrophages.

## Materials and methods

### Preparing flavonoid-based primers

Flavonoids from different subclasses, including Baicalein, Kaempferol, and Naringin, were purchased from Sigma-Aldrich (St. Louis, MO, USA). Based on a previous study [10], the compounds were accurately weighed using an analytical precision balance (Shimadzu, AUW220D, Japan) and dissolved in 20% ethanol. The mixtures were stirred on a magnetic plate for 24 h at room temperature (RT), with the vials shielded from light by wrapping them in aluminum foil, until complete homogenization was achieved, resulting in a final concentration of 20 mM. The solutions were subsequently filtered through a 0.22 μm membrane (Sigma-Aldrich) before their use. Any remaining solution after filtration was discarded. All solutions were freshly prepared immediately before each experiment and were not stored.

### Induction of caries using a microcosm biofilm

Fifty-six sound human third molars were obtained after approval by the local (University of Michigan, Ann Arbor, MI, USA) Institutional Review Board (HUM-00154490). All teeth were cleaned by hand-scaling with a periodontal curette (SS White Duflex, Juiz de Fora, MG, Brazil) and polished with a paste of pumice and water. Afterwards, they were stored in an aqueous solution of 0.5% Chloramine-T (Merck KGaA, Darmstadt, Germany) at 4 °C for no longer than one month. The occlusal enamel was removed using a diamond saw (Buehler Ltd., Lake Bluff, IL, USA), and the dentin at a medium depth relative to the pulp, approximately 2 mm above the cementoenamel junction, was exposed. The dentin surfaces were then polished with 600-grit silicon carbide paper under water-cooling for 5 s to create a flat surface and standardize the smear layer. The enamel of the teeth was coated with nail varnish, leaving only the dentin exposed, and then sterilized in an autoclave at 121 °C for 15 min while immersed in saline solution to maintain tissue hydration. Previous studies have shown that short-cycle moist heat sterilization does not significantly affect dentin morphology nor its chemical composition and has been widely adopted in caries biofilm microcosm models [22, 23].

The caries induction in this study was carried out using a multispecies microcosm biofilm. The protocol for human saliva collection for inoculum preparation followed the methodology described in previous studies [24–26]. A volume of 400 µL of the saliva stock was added to BHI medium (Sigma-Aldrich) without salivary proteins, supplemented with 1% sucrose, and incubated in a CO₂ chamber for 24 h to allow activation. Following this period, the suspension was mixed in a 1:1 ratio with fresh medium, and the dentin specimens were individually placed in a 24-well plate and incubated under identical conditions for an additional 24 h to form the salivary pellicle [26]. On the subsequent day, the growth medium was aspirated, and each specimen was gently rinsed twice with 1 mL of saline solution. A fresh culture medium supplemented with 1% sucrose was added to a new plate. This procedure was repeated daily for seven days until the formation of the artificial dentin carious lesion [25].

### Preparing caries-affected dentin

Following caries induction, the infected and softened dentin layer was selectively removed using a standardized protocol previously described in the literature, i.e., 320-grit silicon carbide abrasive paper for 60 s to remove the outer infected dentin while preserving the underlying caries-affected dentin [27]. Subsequently, 0.5 mm-thick discs were prepared from the carious specimens, and their diameter was reduced to 8 mm using a cylindrical diamond bur with a high-speed handpiece. All thickness adjustments needed to reach the final 0.5 mm were made solely from the pulpal side, using 600-grit silicon carbide abrasive paper under continuous water irrigation, to ensure that the caries-affected surface remained unaltered. The specimens were then adjusted to dimensions suitable for insertion into artificial pulp chambers (APC), which were fixed in the device using rubber rings and wax between the specimen and the ring to seal any space that would allow primer diffusion other than through the dentin.

After the APCs preparation, 0.5 M EDTA (pH 7.4) was applied to both surfaces of the dentin specimens (occlusal and pulpal) for 60 s to remove the smear layer, followed by thorough washing with water [28]. The APC devices with the carious dentin discs were then sterilized using ethylene oxide before the initiation of the cellular experiments [28, 29].

### Cell cultures

Three cell types were employed to comprehensively evaluate the biological effects of the flavonoid-based primers: murine odontoblast-like cells (MDPC-23), human dental pulp stem cells (DPSCs), and murine macrophages (RAW 264.7).

The MDPC-23 cells were selected because they represent a well-established immortalized lineage exhibiting an odontoblast-like phenotype [28, 29]. Cells were maintained in Dulbecco’s Modified Eagle Medium (DMEM, high glucose, GIBCO, Grand Island, NY, USA) supplemented with 10% fetal bovine serum (FBS), 1% penicillin-streptomycin, and incubated at 37 °C in 5% CO_2_. Passages 4 to 8 were used in the experiments.

Human dental pulp stem cells (DPSCs) were obtained from Lonza (Cat. No. PT-5025), a commercially sourced primary DPSC population isolated from human third molars and pre-validated for mesenchymal stem cell markers to study stem cell differentiation. DPSCs were cultured in α-MEM (GIBCO) supplemented with 20% FBS, 1% penicillin–streptomycin, and 2 mM L-glutamine. Cells were incubated at 37 °C in 5% CO_2,_ and passages 3 through 8 were used for the experiments.

RAW 264.7 murine macrophages (ATCC TIB-71) were included to model the innate immune response and evaluate the antioxidant modulation induced by transdentinal extracts. RAW 264.7 cells were maintained in DMEM (GIBCO) supplemented with 10% FBS and 1% penicillin-streptomycin. Cells were incubated at 37 °C in 5% CO_2,_ and passages 5 to 11 were used for the experiments.

### Experimental design

The APC devices containing dentin discs were placed in 24-well plates with the pulpal surface facing upward and hydrated in DMEM (GIBCO) for 24 h. After this period, MDPC-23 cells (3 × 10⁴) were seeded onto the pulpal surface in DMEM supplemented with 10% heat-inactivated fetal bovine serum (FBS, GIBCO), 100 IU/mL penicillin, and 100 µg/mL streptomycin (GIBCO). After 24 h of incubation at 5% CO_2_ and 37 °C, the culture medium was replaced with serum-free DMEM, and the devices were inverted to expose the occlusal surface. The groups treated with 35% phosphoric acid (BA, KA, NA, and SC) had the dentin conditioned for 15 s, followed by rinsing with sterile water and simultaneous aspiration, ensuring that no material came into contact with the culture medium. Subsequently, 20 µl (20 µL) of treatment solutions were passively applied for 60 s [10], exclusively to the occlusal dentin surface of the specimen. Excess primer solution was removed using absorbent paper [10], while only the pulpal surface, with adhered cells, remained in contact with the culture medium [28, 29]. The controls and experimental groups are presented in Table 1.

**Table 1:** Study groups according to the treatment of the caries-affected dentin

Group (Abbreviation)	Treatment (*n* = 8)	Phosphoric acid-etching
Negative control (NC)	Ultrapure water	No
Positive control (PC)	29% aqueous hydrogen peroxide solution	No
Solvent control (SC)	20% ethanol	Yes
Phosphoric Acid (PA)	35% Phosphoric acid	Yes
20 mM Baicalein (BA)	20 mM Baicalein in 20% ethanol	Yes
20 mM Kaempferol (KA)	20 mM Kaempferol in 20% ethanol	Yes
20 mM Naringin (NA)	20 mM Naringin in 20% ethanol	Yes

After the application of dentin treatments, the plates were incubated for an additional 24 h. Following this period, cell viability was assessed for the cells seeded on the pulpal surface, along with microscopic analysis of cell morphology using scanning electron microscopy (Tescan MIRA3 FEG-SEM, Tescan USA Inc., Warrendale, PA, USA). The culture medium that had been in contact with the cells and contained treatment components diffused through the dentin was collected for indirect testing on MDPC-23, DPSC cells, and macrophages (RAW 264.7) cultured in plates. These assays evaluated cell viability, mineralization, and antioxidant potential.

### Viability of MDPC-23 cells seeded on dentin

After 24 h of treatment application and collection of the culture medium containing the extracts, the APC devices with dentin discs were repositioned with the pulpal surface facing upward. The residual culture medium on the pulpal surface was carefully removed to avoid disturbing the adhered cells. A 10% alamarBlue (Invitrogen, Carlsbad, CA, USA) solution was prepared in FBS-free DMEM, and 110 µL of this solution was added to the pulpal surface of the discs. The samples were then incubated for 3 h. Fluorescence intensity was analyzed at 555 and 595 nm (SpectraMax iD3, Molecular Devices, LLC, San Jose, CA, USA). The mean fluorescence value of NC was considered 100% cell viability and used for calculating the cell viability values for the other treatments. For SEM analysis, the APC devices with dentin discs were randomly selected (*n* = 3) and were fixed in a solution of 2.5% glutaraldehyde for 12 h and subsequently dehydrated through a graded ethanol series (30%, 50%, 70%, 90%, and 100%), with each concentration applied for 10 min, followed by two additional immersions in 100% ethanol. After dehydration, the samples were chemically dried using 1,1,1,3,3,3-hexamethyldisilazane (HMDS; Sigma-Aldrich) and left under a fume hood until complete evaporation of the reagent. The specimens were then sputter-coated with gold for SEM analysis (Tescan MIRA3 FEG-SEM).

### Cell response in contact with the extracts obtained from transdentinal diffusion

DPSC and MDPC-23 cells were cultured in 96-well plates (1 × 10⁴/well) on the same day of dentin treatments. The plates were incubated with α-MEM and DMEM with 15% and 10% FBS for the respective cells for 24 h. The next day, 100 µL of extracts were applied to the cells and incubated for 24 h at 5% CO_2_ and 37 °C. Afterward, the extracts were aspirated and replaced by fresh culture medium renewed every 2 days for a total of 7 days.

For cell viability analysis, the alamarBlue protocol was applied to both cells at different time points, as previously described, at 1, 2, and 7 days after contact with the extracts. The mean fluorescence value of NC at day 1 was considered 100% cell viability and used for calculating the cell viability values for the other treatments throughout the time points (paired samples).

The same cells used for cell viability analysis were also evaluated for mineralized nodule formation. For the MDPC-23 cells, mineralization was assessed after 7 days of culture, coinciding with the final viability evaluation. In contrast, for DPSCs, cells were cultured for an additional 7 days in osteogenic medium, resulting in a total of 14 days of culture. The osteogenic medium, corresponding to basal medium supplemented with 100 nM dexamethasone, 10 mM β-glycerol phosphate, and 50 mg/mL ascorbic acid (Sigma-Aldrich), was refreshed every two days throughout the osteoinduction period. After that, the cells were fixed with 70% ethanol and stained with Alizarin red solution. The mineralized nodules were dissolved in cetylpyridinium chloride solution (10 mM, Sigma-Aldrich), and the absorbance was measured at 570 nm. The values were normalized to percentages, considering NC as 100% mineralized nodule deposition. Before dissolving the nodules, representative images of the calcium deposits were taken under a light microscope.

### Antioxidant potential

To evaluate the antioxidant potential of baicalein, one test performed was the Reactive Oxygen Species (ROS) assay. For this test, RAW 264.7 murine macrophage cells (at passage 8) were seeded at a density of 5 × 10⁴ cells/well in 96-well plates were grown in Dulbecco’s Modified Eagle’s Medium (DMEM; GIBCO, Grand Island, NY, USA) supplemented with 10% fetal bovine serum (FBS, GIBCO), 100 IU/mL penicillin, and 100 µg/mL streptomycin (GIBCO) for 24 h at 37 °C and 5% of the CO_2_ environment. Subsequently, the cells were divided into two groups: one exposed to LPS and one without LPS exposure. The culture medium (DMEM) was supplemented with 0.1 µg/mL of LPS and incubated with the cells for 6 h. For the group not exposed to LPS, the culture medium was refreshed. After this incubation period, the medium was replaced with transdentinal extracts, and the cells were incubated for an additional 24 h. Following this, the culture medium was replaced with 100 µL of a solution containing a fluorescent probe for intracellular oxidative stress (Invitrogen, H2DCFDA, Carlsbad, CA, USA) for 10–15 min. Fluorescence intensity was measured with an excitation wavelength of 490 nm and emission at 520 nm (SpectraMax iD3, Molecular Devices, LLC, San Jose, CA, USA).

### Statistical analysis

The independent variable was the dentin treatment, and the primary outcome was the viability of cells seeded on caries-affected dentin discs. The total sample size (*n* = 8 dentin discs per group) was based on previously published transdentinal cytotoxicity studies that demonstrated the ability to detect statistically significant differences among dentin pretreatments under similar experimental conditions [24]. The experiments were conducted in two independent experimental occasions (*n* = 4 per occasion) to ensure internal validity and reproducibility. In all analyses, the dentin disc was considered the experimental unit.

Data were analyzed using GraphPad Prism software version 8.4.3 (GraphPad Software, San Diego, CA, USA), with a significance level set at α = 0.05. The normality of data distribution was assessed using the Shapiro-Wilk test, and the homogeneity of variances was evaluated with Levene’s test. Depending on the experimental design, statistical comparisons were performed using one-way or two-way Analysis of Variance (ANOVA), followed by Sidak’s multiple comparisons test when appropriate. For repeated measurements, such as cell viability assays, data were analyzed using Repeated Measures ANOVA (RM-ANOVA) followed by Sidak’s post hoc test.

## Results

### Viability of MDPC-23 seeded on caries-affected dentin

**Fig. 1:**
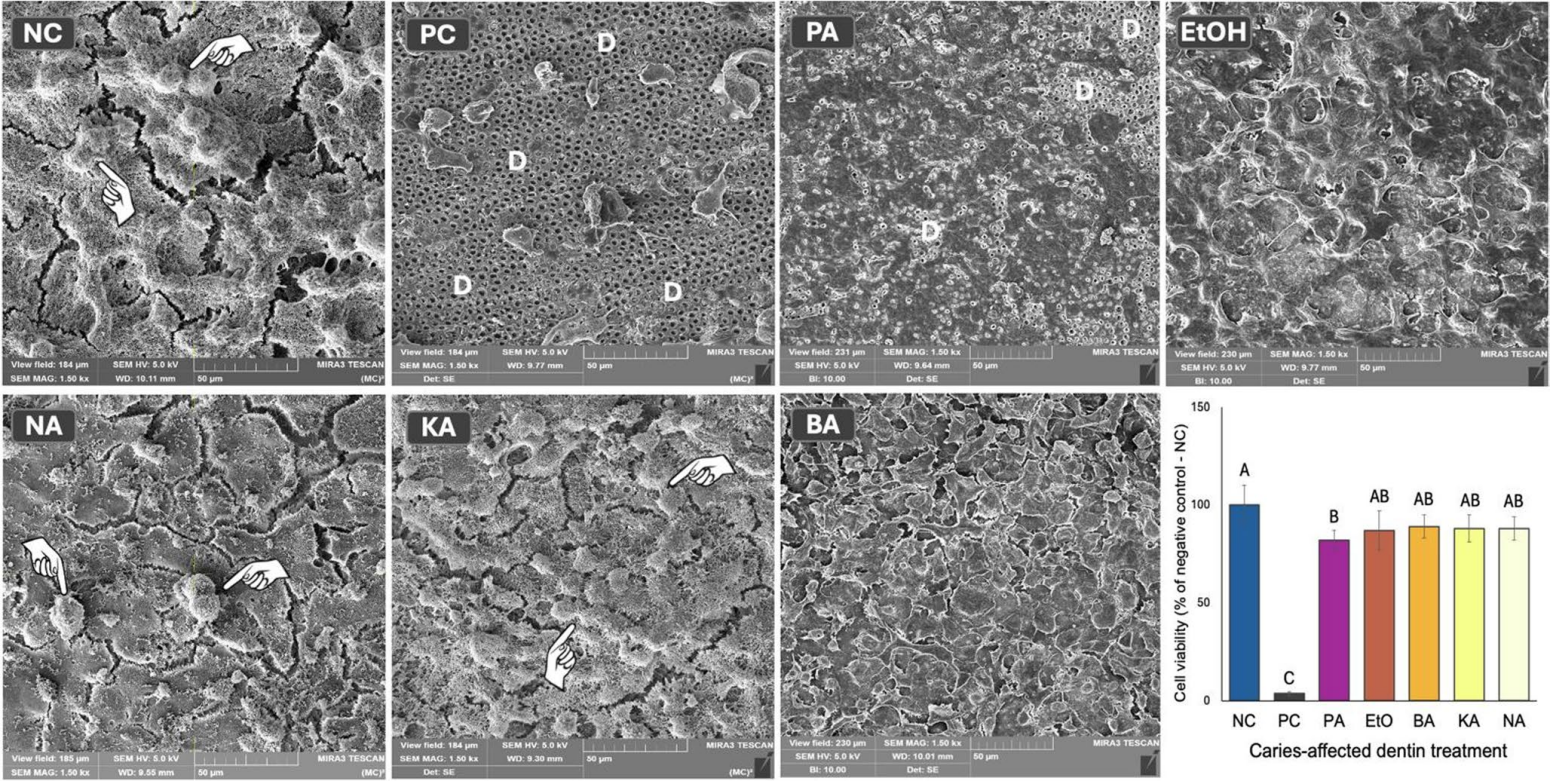
Cell viability of odontoblast-like cells MDPC-23 seeded on caries-affected dentin discs, after 24 h from treatments. Representative SEM photomicrographs (1,500× magnification) showing the morphology of cells that remained attached to the substrate after 24 h from the treatment of phosphoric-etched dentin. In the NC group, the cells cover the entire dentin surface and exhibit a typical morphology, characterized by large, flat cytoplasm and numerous cytoplasmic extensions. On top of these cells, some MDPC-23 cells are undergoing mitosis (indicated by pointers). In contrast, only a few cells remained attached to the dentin in the PC group, exposing large areas of dentin (D). The remaining cells exhibited contracted cytoplasm and lacked projections. After phosphoric acid etching, MDPC-23 lost its typical morphology, and the detachment of some cells exposed areas of dentin (D). The same was seen after the application of ethanol (EtOH), used as a control vehicle, on the etched dentin. The application of the flavonoid-containing primers seems to have contributed to mitigating these adverse effects. For NA and KA, cell number and morphology

The viability of MDPC-23 cells seeded on the pulpal side of caries-affected dentin discs is shown in Fig. 1. The PC group had the lowest cell viability among all tested groups, with a reduction of 97.8% (*p* < 0.0001). Applying 35% phosphoric acid also caused a significant decrease of 17.1% compared to the NC group (*p* = 0.0060). The other groups, SC and flavonoid-based primers, did not differ significantly from the NC and PA groups (*p* ≥ 0.005). Overall, the decrease in cell viability caused by the flavonoid-based primers was less than 15%.

The morphological features of MDPC-23 cells seeded on the pulpal surface of the dentin discs are shown in Fig. 1. In the NC group, a dense and confluent cell layer covered the entire dentin surface, with no dentinal tubules exposed. Typical morphology of MDPC-23 is observed, with cells displaying large, flat cytoplasm and numerous extensions on their surface responsible for attachment to the substrate. Mitotic cells were visible on top of the layer of dentin-attached cells. In contrast, the PC group showed large areas of exposed dentinal tubules with only a few attached cells, which displayed morphological changes such as cytoplasmic contraction, membrane rupture, and a lack of cytoplasmic projections. For the NA- and KA-based primers, cell morphology resembled that of the NC group, while cells with slightly altered characteristics were seen for the BA-based primer. Despite these differences, the cells remained attached to the dentin, with no dentinal tubules exposed. Phosphoric acid caused severe morphological changes in the MDPC-23 cells, leading to areas of exposed dentin due to cell detachment.

### Cell response in contact with extracts

**Fig. 2:**
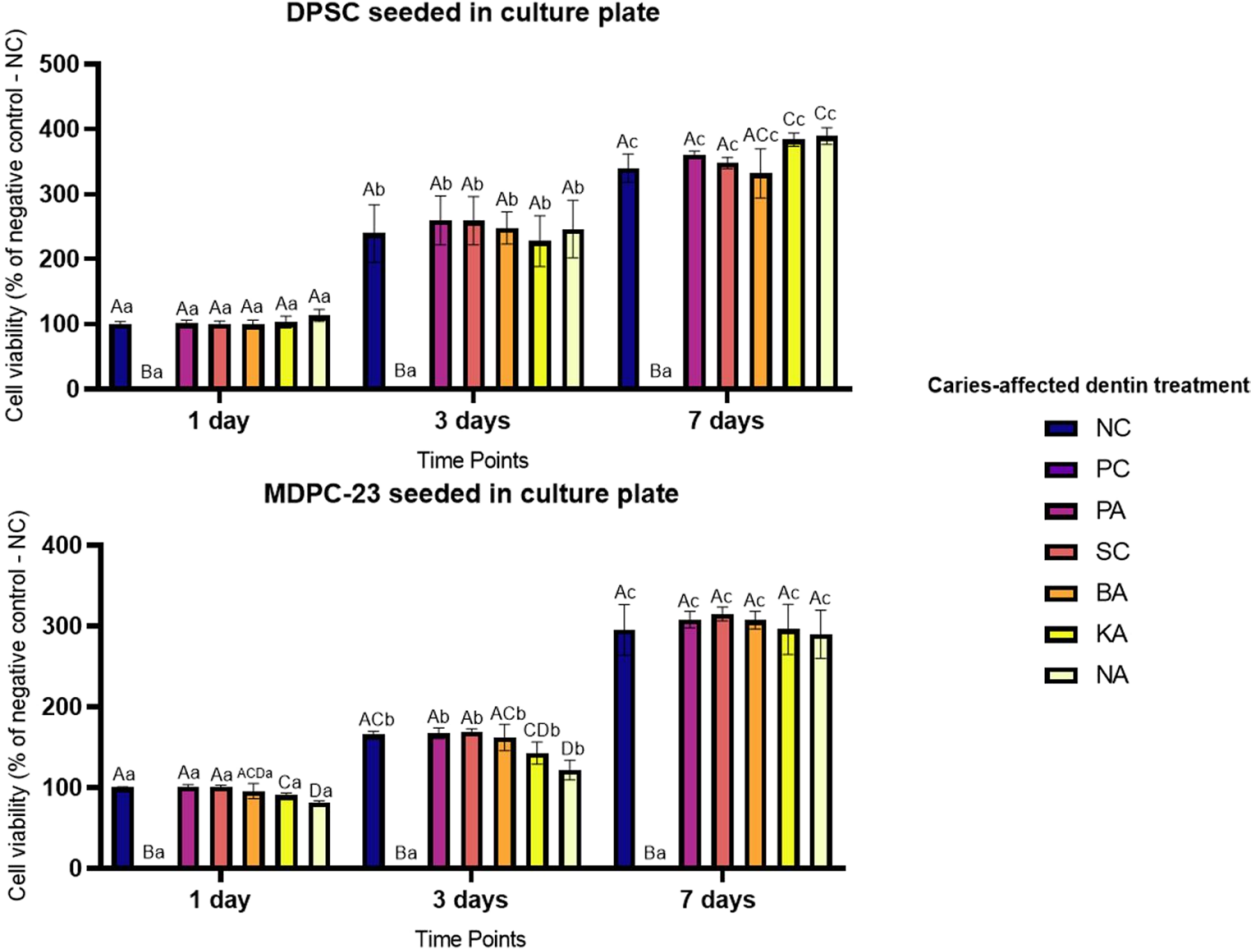
Viability of DPSC and MDPC-23 after contact with the extracts obtained from the transdentinal diffusion of the treatments. Cell viability was assessed at days 1, 3, and 7 after 24-contact with the extracts. Columns represent means, and error bars represent standard deviations of percentages calculated based on the NC group on day 1 as 100%. Uppercase letters compare treatments within the same time point. Lowercase letters compare the same treatment throughout the time point. Different letters indicate statistical significance (RM-ANOVA/Sidak, α = 5%, *n* = 8)

**Fig. 3:**
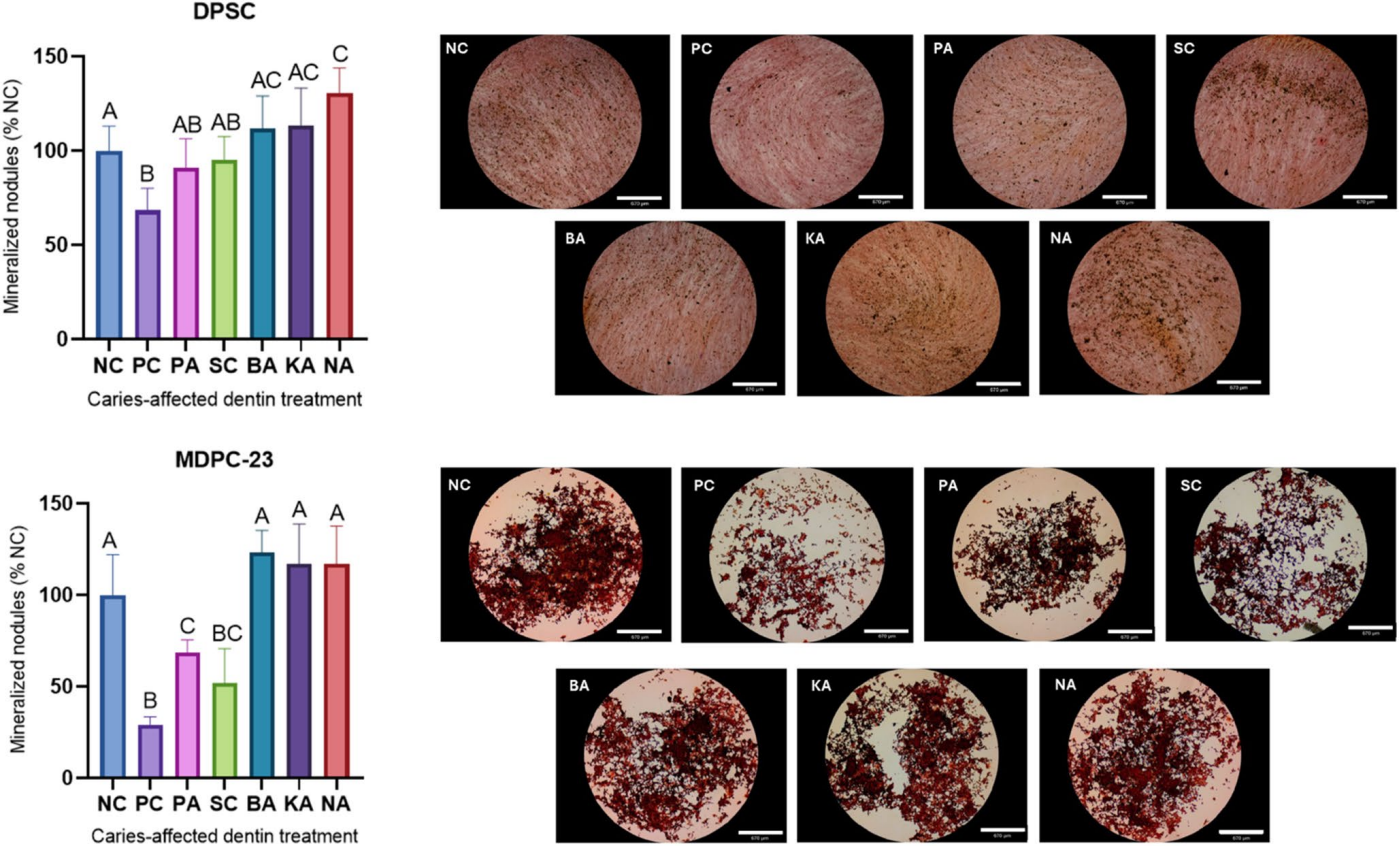
Mineralized nodule formation for DPSC and MDPC-23 cells seeded in plates after 7 days from the 24 h contact with extracts. Different letters indicate statistical significance (One-way ANOVA/Sidak, α = 5%, *n* = 8). Representative images of mineralization nodule deposition are shown to the right of the graphs. Scale bar = 670 μm

The viability and mineralization responses of both cell lines in contact with extracts obtained after transdentinal diffusion of treatments at different time points (1, 3, and 7 days) are shown in Figs. 2 and 3, respectively. For both pulp cell lines, the PC group exhibited a significant reduction in cell viability at all three time points. For DPSC, at 1 and 3 days, none of the experimental groups showed a significant difference compared to the NC (*p* > 0.05). However, at 7 days, the experimental groups treated with KA and NA-based primers showed a significant increase in cell viability (~ 14%) compared to the NC group (*p* = 0.0275 and *p* = 0.0141, respectively). Conversely, the MDPC-23 results revealed distinct response patterns to the primers at different time points. On day one, the experimental primers containing KA (*p* = 0.0057) and NA (*p* < 0.0001) showed a significant reduction in cell viability compared to NC, but less than 20%. On day 3, only the NA group reduced viability compared to NC (*p* = 0.0013). However, after 7 days of exposure, none of the experimental groups showed significant differences in viability compared to the NC (*p* > 0.05).

The analysis of mineralized nodule formation revealed notable differences among the experimental groups in both tested cell types **(**Fig. 3**)**. In DPSCs, NA was significantly different from the NC (*p* = 0.0223). In contrast, BA and KA showed no difference from NA (*p* = 0.5131 and *p* = 0.6872, respectively). However, in the MDPC-23 cells, the PC, PA, and SC groups significantly reduced mineralized nodule formation, while the flavonoid-based experimental primers did not show significant differences compared to the NC group (*p* < 0.0001).

### Antioxidant potential

**Fig. 4:**
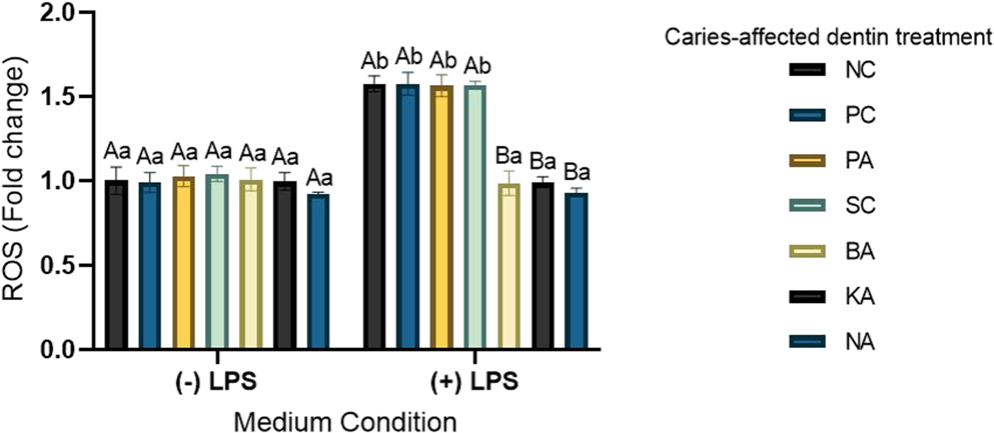
Reactive oxygen species production (fold increase of negative control/-LPS) after 24-hour extract obtained after transdentinal diffusion of treatments applied on macrophages (RAW 264.7). Uppercase letters compare treatments within the same condition of stimulus. Lowercase letters compare the same treatment throughout the condition (-) or (+) LPS. Different letters indicate statistical significance (Two-way ANOVA/Sidak, α = 5%, *n* = 8)

Figure 4 shows that LPS stimulation significantly increased ROS production (*p* < 0.0001). The data are presented as a fold increase over the control (LPS-free medium). In the absence of LPS stimulation, no significant differences were observed among the tested groups compared to the NC (*p* > 0.05). Conversely, under LPS stimulation, all tested flavonoid-based primers significantly reduced ROS to baseline levels compared to the other groups (*p* < 0.0001) and did not differ (*p* > 0.9999) from the condition without LPS stimulation.

## Discussion

The use of natural compounds, especially flavonoids, has been widely studied in developing materials and techniques as alternatives for clinical applications. These compounds have demonstrated potential for modifying the dentin substrate and improving the durability of restorations [10–12]. This study examined the transdentinal response of pulp cells to experimental flavonoid-based primers. An in vitro model was used to assess the biological response of restorative materials applied to dentin, simulating a clinical scenario. The goal was to replicate a situation involving a deep carious lesion with affected dentin, followed by applying flavonoid-based primers to the dentin surface for 1 min, as previously developed by our group [10], under conditions in which the pulp remains vital and is not exposed. In this context, the use of flavonoid-based primers is intended as a dentin biomodification pretreatment in situations where a clinically acceptable remaining dentin thickness is preserved, as approximately 0.5 mm or more is generally required to avoid pulp injury after restoration [30]. Considering that the management of deep cavities remains debated due to principles of selective caries removal, indirect pulp capping, bioactivity of liners and bases, and evidence demonstrating similar long-term clinical success regardless of the material placed [31, 32], the primers evaluated here are not proposed for application directly over areas of imminent pulp exposure or as substitutes for protective liners. Instead, their role is to enhance the quality of the caries-affected substrate by stabilizing the dentin matrix and promoting beneficial biological effects, thus supporting restorative procedures when sufficient residual dentin thickness ensures pulp vitality.

Although previous in vitro studies often used dentin discs with a thickness of 0.4 mm [29], we selected 0.5 mm discs for this study. This choice was made because the dentin samples were caries-affected and thus demineralized, aiming to better simulate clinically relevant conditions. Using a microbiological caries induction protocol further strengthened the transdentinal evaluation method by promoting structural and biochemical changes similar to those seen in natural carious dentin [33]. For instance, it increases permeability due to biochemical alterations, such as higher organic content, particularly degraded collagen [16–18, 33]. The experimental primers were applied passively for 60 s to caries-affected dentin that had been previously etched with 35% phosphoric acid. After application, the primers were not rinsed off; only excess material was removed. This protocol aimed to simulate dentin pre-treatment before adhesive resin application [10]. The results showed that the experimental flavonoid-based primers did not have cytotoxic effects on odontoblastic cells. Consequently, the first null hypothesis, that the primers would not affect pulp cell viability, was accepted.

The controls used in this study were validated in prior research [29, 34]. Ultrapure water was chosen as the NC because it does not cause cytotoxicity in pulp cells, while 29% hydrogen peroxide was used as the PC due to its well-known cytotoxic effects [35, 36]. Although the flavonoid-based primers did not show direct cytotoxicity to odontoblastic cells, a 17.1% decrease in cell viability was observed in the group treated with 35% phosphoric acid compared to the NC. However, this decrease remained below 30%, which, according to ISO 10993-5 (2009) [35] and ISO 7405 (2018) [36], is not regarded as a significant cytotoxic effect.

The cytotoxic effects of phosphoric acid on pulp cells are influenced by various factors, including cavity depth and, most importantly, direct and prolonged contact with dentin [37, 38]. Since the dentin in this study was already demineralized by caries, the cytotoxic effects of phosphoric acid were likely heightened. Additionally, there was no simulation of intrapulpal pressure, which could dilute the acid and prevent it from reaching the cells or alter the pH of the culture medium. To ensure consistency, phosphoric acid was applied to all experimental groups (BA, KA, and NA). Cell viability in groups treated with flavonoid-based primers showed no significant differences compared to the phosphoric acid-treated group. However, phosphoric acid did modify MDPC-23 morphology. Cells displayed contracted cytoplasm with the loss of cytoplasmic extensions. However, they remained attached to the dentin **(**Fig. 1**)**. Since the morphology of MDPC-23 cells treated with flavonoid primers resembles that of the negative control (ultrapure water), it is plausible to hypothesize that the activity of the flavonoids counteracted the negative effects of the phosphoric acid.

The flavonoid concentration used to formulate the primer solutions (20 mM) was based on previous research [10], which showed an improvement in dentin-resin bond stability over time compared to lower concentrations. Additionally, other studies investigating flavonoid-based solutions as dentin pre-treatment agents have also used high concentrations of these compounds [12]. However, results on flavonoid toxicity in cells suggest that even very low concentrations (10–20 µM) can cause adverse effects [19]. In this study, extracts collected from the primer solutions 24 h after application were tested at different exposure times (1, 3, and 7 days). For DPSC cells, no significant differences were observed among groups in the initial days. However, after 7 days, the KA- and NA-treated groups showed increased cell viability compared to the NC, which is important for clinical use. Conversely, for MDPC-23 cells, KA and NA reduced cell viability by about 14% at 1 and 3 days, but this reduction did not reach cytotoxic levels. After 7 days, no significant differences were seen between the experimental groups and the NC. Despite using a high flavonoid concentration (ca. 1,000 times higher than the threshold for cytotoxicity in some studies), it appears that flavonoids mostly bind to collagen through non-covalent interactions, acting as crosslinking agents [39, 40]. This interaction may have limited their diffusion into the dentin tissue, and the presence of demineralized dentin likely enhanced their binding efficiency. Furthermore, the demineralized tissue may have served as a semi-permeable barrier, functioning as a physical and biochemical filter that restricted the passage of high concentrations of the solutions, thereby protecting the underlying dental pulp cells.

The molecular structure of flavonoids varies depending on the presence and amount of hydrogen bonds, aromatic rings, hydroxyl, amine, and carbonyl groups, as well as double bonds between carbon atoms [14]. These structural differences directly affect their interaction with tissues and cells. In this study, the increased viability of DPSC cells after 7 days of exposure to KA and NA extracts may be linked to the specific structural features of these flavonoids. For example, these flavonoids contain more hydroxyl (OH) groups, conjugated double bonds, and have a different arrangement of rings A and B compared to baicalein [14]. These structural differences can influence their effectiveness. Although both compounds have been reported to promote cell proliferation [41, 42], studies show that kaempferol and naringin activate cellular signaling pathways such as phosphoinositide 3 kinase (PI3K/Akt) and Wnt/β-catenin [41, 43], which are directly involved in cell proliferation. In contrast, baicalein exhibits superior anti-inflammatory and antioxidant properties [44] and may also affect the differentiation of mesenchymal stem cells [45], which are precursors of pulp cells.

Beyond the Wnt/β-catenin pathway, kaempferol and naringin also activate osteogenic and odontogenic signaling pathways associated with cell differentiation. Among these, bone morphogenetic protein-2 (BMP-2) is a key regulator of osteogenesis, while runt-related transcription factor 2 (Runx2) is essential for mineralized matrix formation [21, 42, 46]. Additionally, the Wnt/β-catenin pathway itself plays a fundamental role in the development of mineralized tissues [21]. Naringin has also been shown to upregulate mineralization-related genes, enhancing calcium deposition and hydroxyapatite crystal formation by stimulating osteocalcin and alkaline phosphatase via the BMP-2 and extracellular signal-regulated kinase (ERK) pathways [47, 48]. This may explain the significant differences observed in mineralized nodule formation among the tested groups, with NA exhibiting the most significant effect. Therefore, the second null hypothesis, i.e., that the experimental primers would not promote the formation of mineralization nodules, was rejected. However, this increase in nodule formation was only observed in DPSC cells, while no flavonoid was able to enhance mineralized matrix formation in MDPC-23 cells. This may be attributed to the fact that DPSC cells are stem cells that respond to proliferative signals induced by flavonoids via pathways such as PI3K/Akt, Wnt/β-catenin, and Runx2 [21, 48, 49], whereas MDPC-23 cells are more differentiated and less responsive to growth-promoting stimuli.

The anti-inflammatory and antioxidant properties found in baicalein are also present in kaempferol and naringin, due to their common structural feature of an aromatic ring with C2-C3 unsaturation, where the position of the hydroxyl group is crucial for these functions [14]. Flavonoids exert these effects through various mechanisms, including scavenging free radicals, modulating antioxidant enzymes, and inhibiting reactive oxygen species (ROS) [50–52]. An imbalance of ROS can lead to oxidative stress, inflammation, and degenerative diseases [52, 53], which is especially relevant in deep caries lesions where bacterial invasion and endotoxin diffusion into the dentin-pulp complex worsen oxidative and inflammatory processes [54]. Interestingly, flavonoids play a dual role in ROS homeostasis: acting as antioxidants under normal conditions, while triggering apoptotic pathways in cancer cells due to their strong pro-oxidant activity [52]. In this study, all tested flavonoids effectively lowered ROS levels when applied to LPS-stimulated macrophages, mimicking an inflammatory state. Therefore, the third null hypothesis was also rejected.

Based on the findings of this study, flavonoid-based experimental primers can be considered a safe option for dentin pre-treatment, even in deep cavities and caries-affected dentin, as they demonstrate cytocompatibility with pulp cells. One limitation of this study is the lack of quantification of residual flavonoids in the extracts after primer application due to potential interactions with culture medium and metabolites. Additionally, dentin permeability and pulpal fluid dynamics could influence the clinical outcome of these primers. Therefore, additional in vivo and clinical studies are important to validate these findings and to compare them with the beneficial effects previously observed in laboratory studies.

## Conclusion

The experimental flavonoid-based primers can be used as dentin pretreatment on caries-affected dentin and in deep cavities, as they did not show cytotoxicity to pulp cells. Additionally, these primers offer extra benefits, including stimulating mineralization by pulp cells, and reducing reactive oxygen species by macrophages, which might help regenerative-related processes within the dentin-pulp complex. This supports their potential as a dentin pre-treatment in dental restorative procedures.

## Data Availability

All data generated or analyzed during this study are included in this published article.
